# Systemic leptin produces a long-lasting increase in respiratory motor output in rats

**DOI:** 10.3389/fphys.2013.00016

**Published:** 2013-02-12

**Authors:** Zheng Chang, Edmund Ballou, Weijie Jiao, Kevin E. McKenna, Shaun F. Morrison, Donald R. McCrimmon

**Affiliations:** ^1^Department of Physiology, Feinberg School of Medicine, Northwestern UniversityChicago, IL, USA; ^2^Department of Neurological Surgery, Oregon Health and Science UniversityPortland, OR, USA

**Keywords:** leptin stimulation of breathing, neural control of breathing, metabolic control of breathing, leptin, respiratory modulation

## Abstract

Leptin decreases food intake and increases energy expenditure. Leptin administration into the CNS of mice or rats increases alveolar ventilation and dysfunction in leptin signaling has been implicated in the hypoventilation that can accompany obesity. An increase in CO_2_ chemosensitivity has been implicated in this response but it is unclear whether ventilation is augmented when PCO_2_ is maintained constant. We examined the effects of intravenous leptin to test the hypothesis that systemic leptin administration in isoflurane anesthetized, mechanically ventilated and vagotomized rats would lead to a sustained increase in respiratory motor output that was independent of changes in end-tidal PCO_2_, body temperature or lung inflation pressure (an indicator of overall lung and chest wall compliance). In anesthetized Sprague-Dawley rats with end-tidal PCO_2_, lung compliance and rectal temperature maintained constant, injection of a bolus of leptin (0.25 mg, 0.5 mg/ml, i.v.), followed over the next 1 h by the intravenous infusion of an additional 0.25 mg, elicited a progressive increase in the peak amplitude of integrated phrenic nerve discharge lasting at least 1 h beyond the end of the infusion. The increase peaked at 90 min at 58.3 ± 5.7% above baseline. There was an associated increase in the slope of the phrenic response to increasing inspired CO_2_. There was also a moderate and sustained decrease in arterial pressure 9 ± 1.3 mmHg at 120 min, with no associated change in heart rate. These data indicate that leptin elicits a sustained increase in respiratory motor output that outlasts the administration leptin via a mechanism that does not require alterations in arterial PCO_2_, body temperature, or systemic afferent feedback via the vagus nerves. This stimulation may help to prevent obesity-related hypoventilation.

## Introduction

The cytokine, leptin, is an anorexogenic hormone that plays an important role in the regulation of energy balance. Administration of leptin reduces eating and increases energy expenditure (Cone, 2005; Myers et al., 2008; Cammisotto et al., 2010) largely through a sympathetically mediated increase in brown adipose thermogenesis (Morrison, 2004) and through an increase in spontaneous physical activity (Choi et al., 2008).

Adipocytes are a major source of leptin and obese humans have substantially elevated circulating leptin levels, consistent with their high percentage of body fat. The increased metabolic activity associated with obesity increases CO_2_ production, necessitating a corresponding increase in alveolar ventilation to maintain homeostasis of arterial PCO_2_ (PaCO_2_) and acid-base status. A subset of obese individuals hypoventilate with substantial elevations in PaCO_2_ in a condition termed obesity hypoventilation syndrome (Zwillich et al., 1975; Mokhlesi et al., 2008; Mokhlesi, 2010; Piper and Grunstein, 2011). The failure of the high leptin levels to suppress appetite and increase energy expenditure in these obese individuals is generally attributed to a resistance to leptin (Myers et al., 2008; Al Dabal and BaHammam, 2009).

Studies in mice are consistent with the concept that leptin is an important contributor in the normal linkage between metabolism and alveolar ventilation in obesity. For example, adult ob/ob mice have a mutation in the gene for leptin and lack functional leptin, are markedly obese and hypoventilate with consequent marked increases in PaCO_2_ (PaCO_2_ > 45 mmHg). Associated with the hypoventilation is a decrease in chemosensitivity as indicated by a reduction in the slope of the relationship between minute ventilation and PaCO_2_ (O'Donnell et al., 1999). Administration of leptin either intravenously or into the cerebral ventricles of these mice restores chemosensitivity and normocapnia (O'Donnell et al., 1999; Bassi et al., 2012).

The potential role of leptin in respiratory control has also been examined in rats, but the outcome is less clear. Obese Zucker or Koletsky rats with mutations in the receptor for leptin exhibit abnormal breathing patterns (Farkas and Schlenker, 1994; Strohl and Thomas, 2001), but a heritability analysis of the breathing pattern abnormalities in Zucker rats only attributed a minor role to the leptin receptor (Iyengar et al., 2004). Nevertheless, injections of leptin directly into the nucleus of the solitary tract (NTS) produce a substantial, dose-dependent stimulation of breathing, primarily through an increase in the magnitude of phrenic nerve bursts, the neural equivalent of tidal volume (Inyushkin et al., 2009; Inyushkina et al., 2010). The reasons are not clear for this apparent discrepancy in the linkage between leptin and alveolar ventilation. One possibility is that obese Zucker and Koletsky rats develop compensatory changes in respiratory control that make them less dependent on leptin for respiratory homeostasis. Additionally, a number of factors are difficult to control for, such as the impact of obesity on pulmonary mechanics, metabolism, and thermoregulation and on the regulation of arterial blood gases and acid-base balance.

The current study was designed to test the hypothesis that systemic leptin administration in rats would lead to a sustained increase in respiratory motor output that was independent of leptin-induced changes in CO_2_ production, body temperature or overall lung mechanics. The findings support the concept that when end-tidal PCO_2_, body temperature, and overall lung mechanics are maintained within narrow limits, intravenous leptin administration produces a time-dependent and sustained increase in respiratory motor output that outlasts the period of leptin administration by at least 60 min.

## Materials and methods

### Animals

Experiments were performed on adult male Sprague-Dawley rats (Charles River, Wilmington, MA, USA) weighing 300–500 g. All surgeries were performed using sterile procedures adapted for small rodents, in accordance with guidelines recommended by the NIH and by the Society for Neuroscience. The Northwestern University Animal Care and Use Committee approved all procedures.

### Surgery

Anesthesia was induced with 5% isoflurane (in 50% O_2_, balance N_2_) in an induction chamber. Animals were then rapidly switched from the induction chamber to a nose cone attached to a stereotaxic frame where the animal was allowed to freely breathe isoflurane (2.5–3%) for maintenance. The depth of anesthesia was frequently assessed (10–15 min intervals) and judged by the absence of retraction responses to a strong noxious paw pinch, and by the absence of changes in heart rate or breathing pattern in response to the noxious stimulation. Rectal temperature was monitored and maintained at 37.5 ± 0.5°C by means of a thermistor-controlled heat lamp. The ECG was continuously monitored using transcutaneous needle electrodes placed on the caudal thorax with a ground wire placed laterally on the abdomen. These electrodes also recorded diaphragm EMG prior to paralyzing the animals (see below). Oxygen saturation as well as heart rate and respiratory rate were monitored via a pulse oximeter (Mouse Ox, Starr Life Sciences, Oakmont, PA, USA).

Vagi were sectioned bilaterally in the neck. The trachea was cannulated high in the neck and rats were mechanically ventilated (2.0–2.5 ml, ~70 min^−1^) and airway pressure was continuously monitored. End-tidal PCO_2_ was continuously sampled at the mouth using a rapidly responding infrared CO_2_ monitor (Puritan–Bennett Co., Datex 223) modified to minimize the dead space volume. The tidal volume was increased for 2–3 breaths every 10–15 min to prevent atelectasis.

With these ventilator settings, baseline ventilation was sufficient to lower PCO_2_ below the apneic threshold and CO_2_ was added to the inspired port of the ventilator to raise the end-tidal PCO_2_ 3–5 mmHg above the apneic threshold. The level of inspired CO_2_ was then continuously adjusted to maintain end-tidal PCO_2_ within 1 mmHg of this initial level. Since the administration of leptin increases brown adipose thermogenesis and hence PCO_2_ (Morrison, 2004), for rats receiving leptin the level of inspired CO_2_ was decreased to maintain a constant end-tidal PCO_2_.

The left femoral artery was cannulated (PE-50) for recording arterial pressure. The femoral veins were cannulated (PE-10) bilaterally. One vein was used for infusion of fluids (lactated Ringer's, 3–8 ml/h) and administration of a paralytic, either pancuronium bromide (1 mg/kg/h) or succinyl choline chloride (5 mg/kg/h). The contralateral vein was used for infusion of leptin.

Animals were placed in a stereotaxic frame and a phrenic nerve was exposed in the neck, cleaned of connective tissue, placed on a bipolar silver hook recording electrode and immersed in mineral oil to prevent drying. Nerve activity was amplified (bandpass filtered, 0.3–3 kHz), digitized at 10 kHz and recorded on a laboratory computer using Spike 2 software (CED Ltd., Cambridge, England).

### Experimental protocol

At least 1 h following surgery, baseline levels of integrated phrenic nerve activity, arterial pressure, heart rate, end-tidal PCO_2_, airway pressure, and rectal temperature were recorded for 10–30 min. Leptin was dissolved in lactated Ringer's solution and administered using a protocol similar to that reported by Haynes et al. (1997) with the total dose being within the range that has previously been shown to induce Fos-immunoreactivity in the CNS of adult rats (Elias et al., 2000). Separate groups of rats received leptin (*n* = 6) or lactated Ringer's solution (*n* = 4) intravenously. In the leptin group, an initial bolus of leptin (0.25 mg, 0.5 mg/ml) was administered, followed by the continuous intravenous infusion of an additional 0.25 mg over the following 60 min. The control group received lactated Ringer's (the leptin vehicle) delivered as a 0.5 ml bolus and subsequent 0.5 ml infusion over a 1 h period. Phrenic nerve activity, arterial pressure, heart rate, end-tidal PCO_2_, rectal temperature, and airway pressure were continuously monitored during the pre-injection control and for an additional 1 h following the termination of the infusion. At the end of this recording period, a CO_2_ response curve was generated by increasing the fraction of CO_2_ in the inspired gas. CO_2_ was elevated in two steps; the first was 20–30 mmHg above baseline while the 2nd raised end-tidal PCO_2_ to about 100 mmHg. Each step was maintained for about 4 min with quasi steady-state measurements made during the last 30 s of each step.

Rats were then sacrificed by increasing the inspired isoflurane to 5% followed by a bilateral pneumothorax or decapitation.

### Data analysis

Data obtained during control measurements and for the 2 h test of the effect of leptin administration were analyzed using a nested analysis of variance model with group as the between-rat factor and time as the within rat factor. The group by time interaction term was also tested. *Post-hoc* comparisons were made using Bonferroni-corrected *t*-tests.

Slopes of the CO_2_ response curves for each rat were calculated using a least squares regression through the three levels of CO_2_. Slopes for leptin treated vs. control rats were then compared using a one-tailed *T*-test.

The possible correlation between any change in phrenic nerve activity and a corresponding change in arterial pressure was determined using a Spearman's rank correlation coefficient. Values of phrenic nerve discharge and mean arterial pressure (MAP) during control and at 15 min intervals for 2 h after the initial administration of leptin were compared.

## Results

**Figure 1:**
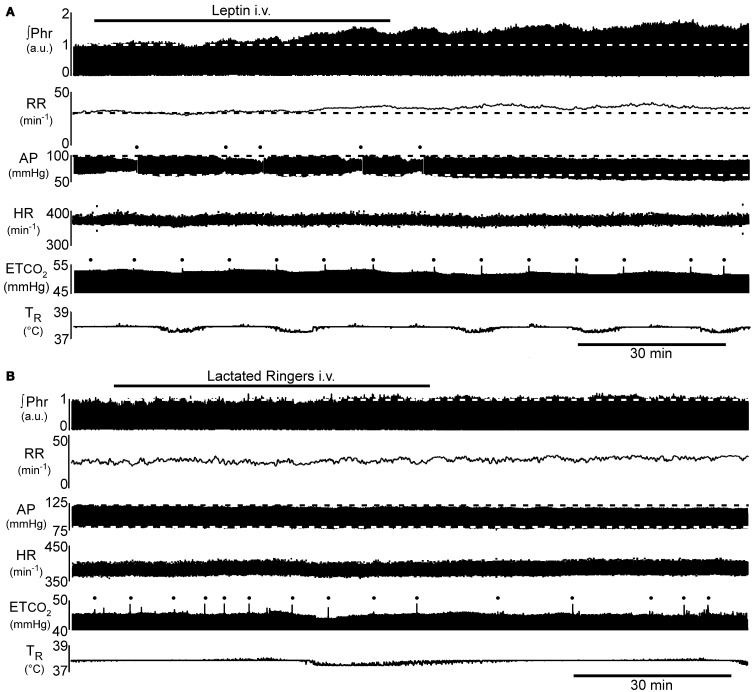
**Example of the time course of the increase in phrenic nerve activity (∫Phr) and decrease in arterial pressure (AP) in response to intravenous leptin. (A)** 250 μg leptin bolus at beginning of the bar indicating leptin injection, followed by 250 μg infused over the indicated period. **(B)** Control response to injection of the vehicle for leptin (lactated Ringer's). Dots above AP trace in **(A)** indicate flush of the arterial line. Dots above end-tidal CO_2_ trace (ETCO_2_) indicate artifacts arising from augmented inflations to prevent atelectasis. a.u., arbitrary units; AP, arterial pressure; ETCO_2_, end-tidal carbon dioxide; HR, heart rate; ∫Phr, integrated phrenic nerve activity; RR, respiratory rate; T_R_, rectal temperature.

Intravenous administration of murine leptin to anesthetized adult rats (*n* = 6) elicited a sustained increase in phrenic nerve discharge and a modest decrease in arterial pressure. Over the course of an experiment, homeostatic parameters that could influence either gas exchange or cardiorespiratory control were maintained within narrow limits for both leptin and control groups of animals. Specifically, no significant changes were recorded in end-tidal PCO_2_, rectal temperature (Figure 1, Table 1), or airway pressure (not shown) over the recording period. Although there was an initial difference in PCO_2_ between the groups, the PCO_2_ of individual animals was maintained within 1 mmHg of its starting value and therefore did not significantly contribute to the progressive increase in phrenic nerve discharge seen in the leptin group (see below). At the beginning of each experiment the ventilator was set at a tidal volume of ~2.5 ml and 70 cycles/min. For both groups CO_2_ was added to the inspired gas mixture to bring the end-tidal PCO_2_ to 3–5 mmHg above the apneic threshold.

**Table 1:** **Body weight, rectal temperature and end-tidal PCO_2_ at 30 min intervals**.

**Group**	**Weight (gms)**	**Rectal temperature (°C) (*n*)**					**End-tidal PCO_2_ (mmHg) (*n*)**				
		**0 min**	**30 min**	**60 min**	**90 min**	**120 min**	**0 min**	**30 min**	**60 min**	**90 min**	**120 min**
Leptin	371.5 ± 11.8 (6)	37.8 ± 0.1 (6)	37.8 ± 0.1 (6)	37.9 ± 0.1 (6)	37.8 ± 0.1 (6)	37.8 ± 0.1 (5)	48.8 ± 2.5 (6)	48.8 ± 2.6 (6)	48.8 ± 2.6 (6)	49.1 ± 2.6 (6)	46.0 ± 2.4 (4)
Control	352.3 ± 16.8 (4)	37.6 ± 0.2 (4)	37.6 ± 0.2 (4)	37.7 ± 0.1 (4)	37.7 ± 0.1 (4)	37.7 ± 0.1 (4)	38.7 ± 3.8 (4)	38.5 ± 3.7 (4)	38.7 ± 3.6 (4)	38.7 ± 3.6 (4)	34.3 ± 6.7 (2)

*The decrease in PCO_2_ at 120 min was due to the loss of 2 rats in each group. There was no change in individual animal PCO_2_. Values are mean ± s.e.m*.

### Respiratory motor responses to intravenous leptin

**Figure 2:**
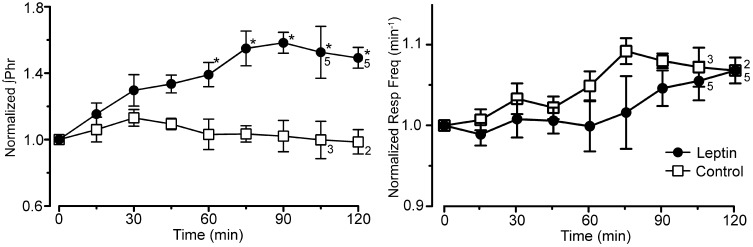
**Integrated phrenic nerve amplitude (∫Phr normalized to baseline) and burst frequency (Resp Freq) at baseline (0 min) and at 15 min intervals for the experiment in Figure 1 (leptin; *n* = 6 except as indicated on graph; control, lactated Ringer's; *n* = 4 except as indicated).** Note the selective increase in ∫Phrenic in leptin treated rats, ^*^p < 0.05.

Injection of a bolus of leptin (250 μg, i.v.), followed over the next hour by the intravenous infusion of an additional 250 μg, elicited a progressive increase in phrenic nerve discharge (Figures 1A, 2; *n* = 5 − 6; *p* < 0.05) lasting at least 1 h beyond the end of the infusion. In contrast, intravenous injection of the vehicle (lactated Ringer's containing phosphate buffered saline; Figures 1B, 2; *n* = 2 − 4) elicited no significant change in respiratory motor output.

The increase in phrenic nerve discharge in leptin treated rats was primarily seen as an increase in the peak amplitude of the integrated phrenic nerve discharge (Figure 2). The increase began within 15 min of the initial leptin injection and was significant at 60 min following the initial injection, at which time the increase averaged 39.2 ± 9.3% above baseline. The increase peaked at 90 min (i.e., 30 min after terminating the leptin infusion) when it averaged 58.3 ± 5.7% above baseline. One hour after terminating the leptin infusion, the peak amplitude of integrated phrenic nerve activity was still elevated 47.5 ± 6.7% above baseline. The neural equivalent of minute ventilation (phrenic burst frequency *x* peak amplitude of each phrenic burst) was also significantly increased from baseline at 75, 90, and 120 min after the initiation of the leptin injection (not shown), although there was no significant change in the phrenic nerve burst frequency (Figures 1A, 2).

**Figure 3:**
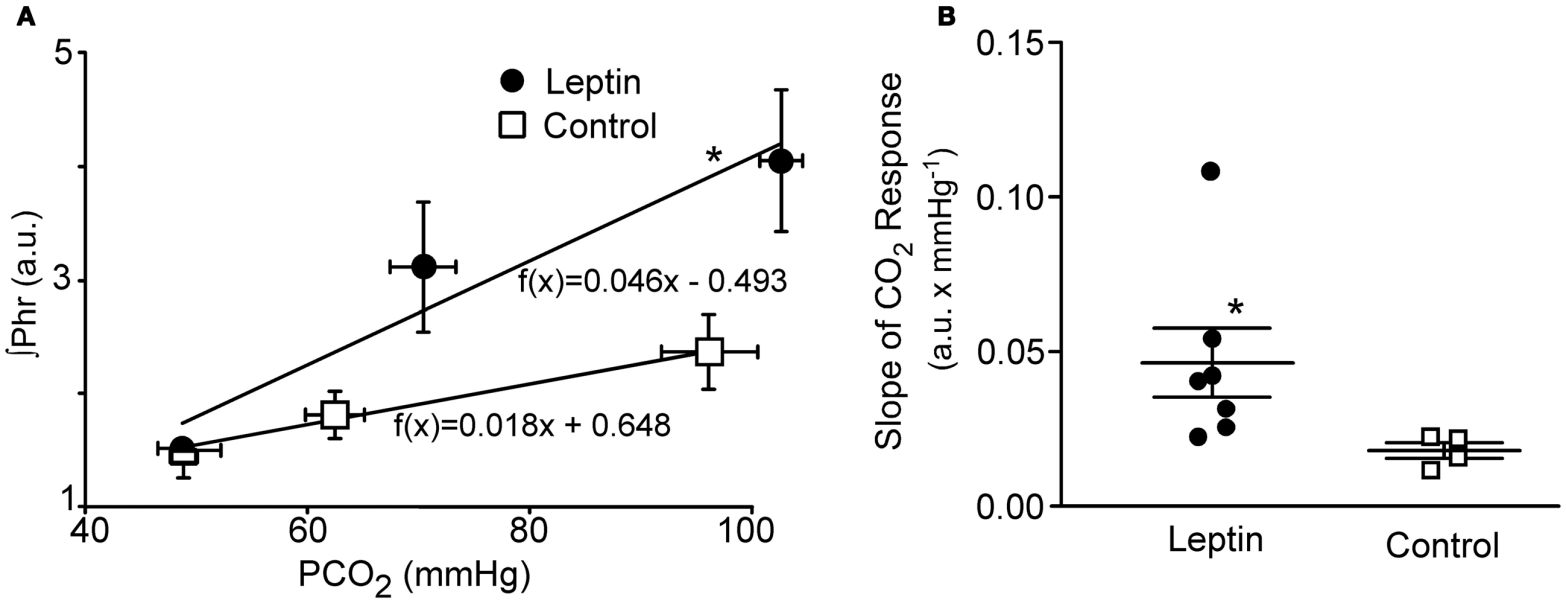
**CO_2_ Response curves in leptin (*n* = 7) vs. control (*n* = 4) rats measured 2–2.5 h following the initial bolus injection of leptin.** Three levels of CO_2_ were delivered via altering the fraction of CO_2_ in the inspired gas delivered via the ventilator. **(A)** Plot of the average phrenic nerve activity at each level of end-tidal PCO_2_. Responses were fit with a least squares regression for which the equations are shown. **(B)** Scatter plot of slopes of the individual CO_2_ responses obtained for each animal as well as the group means ± S.E.M. ^*^P < 0.05. Horizontal error bars in **(A)** indicate differences in the end-tidal PCO_2_ for individual animals.

To test the aggregate sensitivity of central and peripheral chemoreceptors, a CO_2_ response curve was generated at the end of the experiment in both the leptin and control groups of animals. The average slope of the CO_2_ response curve in leptin treated rats (Figure 3; *n* = 7) was more than twice that of control animals (*P* < 0.05; *n* = 4).

### Cardiovascular responses to intravenous leptin

**Figure 4:**
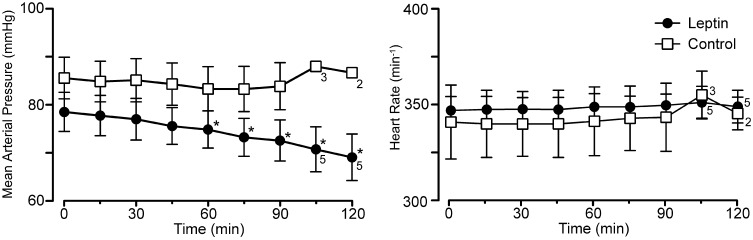
**Mean arterial pressure and heart rate at baseline (0 min) and at 15 min intervals for the experiment in Figure 1 (leptin; *n* = 6 except as indicated on graph; control, lactated Ringer's; *n* = 4 except as indicated).** Note the selective decrease in arterial pressure, ^*^p < 0.05.

Leptin treatment elicited a progressive decrease in MAP over the 120 min following the initiation of leptin administration. At 120 min, MAP had decreased 10 ± 1.3 mmHg from a control of 79 ± 4.1 mmHg (Figures 1, 4; *p* < 0.05). Both systolic and diastolic pressures exhibited similar decreases. There was no significant change in MAP of control animals; compared to an initial value of 86 ± 4.3 mmHg, MAP was 84 ± 4.9 mmHg at 90 min, (*n* = 4) and in the two rats measured at 120 min it was 87 ± 1.0 mmHg (Figures 1, 4). Despite the decrease in MAP in leptin treated rats, there was no change in heart rate over the course of the experiment in either the leptin or control group of animals (Figures 1, 4).

### Correlation between the increase in phrenic discharge and decrease in mean arterial pressure

The Spearman's rank correlation coefficient revealed a significant correlation between the increase in phrenic discharge and decrease in MAP (−0.8833, *P* < 0.05). While the decrease in MAP could contribute to the increase in phrenic discharge, the decrease was relatively small, averaging 10 ± 1.3 mmHg. At the end of the experiment, the MAP was still 69 ± 4.8 mmHg in leptin-treated rats. This is still above the value generally taken as the lower limit for autoregulation (e.g., cerebral autoregulatory range: 60–150 mmHg; Paulson et al., 1990). Thus, a significant decrease in organ perfusion, including the brain, would not be anticipated. Further, equivalent decreases in MAP induced with i.v. sodium nitroprusside in a separate group of rats did not elicit significant increases in phrenic burst amplitude (not shown). Perhaps more importantly, is the progressive nature of the changes in both MAP and phrenic burst amplitude. For instance, if there was a causal relationship, the small decreases in MAP at 30 min (−1.5 ± 0.7 mmHg) and 60 min (−3.7 ± 0.5 mmHg) would have been responsible for 31.7 ± 10.0% and 39.4 ± 8.2% increases in phrenic nerve discharge, respectively. Thus, although a contribution of the decrease in arterial pressure to the increase in the increase in phrenic nerve discharge cannot be completely ruled out, a direct effect of leptin on central respiratory control seems likely to be more important.

## Discussion

The major finding of the current investigation was that systemic leptin infusion causes a progressive and sustained increase in respiratory motor output in isoflurane-anesthetized rats. The average peak amplitude of integrated phrenic nerve bursts increased to almost 160% of baseline 90 min following the initiation of an intravenous leptin infusion. There was little or no associated change in the phrenic nerve burst frequency. There was also a moderate and sustained decrease in arterial pressure, with no associated change in heart rate.

### Critique of methods

An important consideration in the interpretation of the current data is whether PaCO_2_ was constant throughout the test period. A systematic elevation in PaCO_2_ over the course experiment could compromise the data interpretation. In the current study, end-tidal PCO_2_ was used to estimate PaCO_2_. Several steps were taken to optimize the measurement of end-tidal PCO_2_. First, we minimized the dead space of the CO_2_ monitor and the associated sampling line. To reduce the impact of changing ventilator settings on the CO_2_ sampling, we used a relatively large tidal volume (~2.5 ml). To permit fine tuning of the end-tidal PCO_2_ throughout the experiment, the ventilator frequency was increased, prior to beginning leptin administration, sufficiently to lower PCO_2_ several mmHg below the apneic threshold. The F_I_CO_2_ was then increased to raise PCO_2_ 3–5 mmHg above the apneic threshold. Once the ventilator volume and frequency were set at the beginning of the experiment they were not adjusted throughout the remainder of the experiment. This prevented any change in ventilator settings from influencing the end-tidal PCO_2_ measurement. Any adjustment necessary to maintain a constant end-tidal PCO_2_ was accomplished by fine tuning the F_I_CO_2_. To prevent atelectasis, the rats are “sighed” every 10–15 min (e.g., see Figure 1). We also continuously recorded airway pressure to ensure that there were no overall changes in lung mechanics. During control, the inflation pressure for the six leptin treated animals was 7.6 ± 0.5 cmH_2_O (*n* = 6). At the end of the experiment (2 h after beginning leptin infusion) airway pressure was 7.9 ± 0.5 cmH_2_O (*P* = 0.6). In addition, pulse oximetry indicated that O_2_ saturation remained above 95%, suggesting that gas exchange was not impaired over the course of the experiment. Thus, while it is possible that there was a slight difference between our measured end-tidal PCO_2_ and PaCO_2_, we believe any such difference was small. More importantly, any difference between end-tidal PCO_2_ and PaCO_2_ should have been constant throughout the experiment. Hence, we believe that PaCO_2_ was maintained within 1 mmHg of the starting value throughout the experiment.

### Effect of systemic leptin infusion on phrenic nerve activity

Our observation of a leptin-induced increase in phrenic nerve activity in the rat is consistent with earlier work in mice. Mice with a mutation in the *ob* gene do not have leptin, are obese and hypoventilate in all sleep-wake states (O'Donnell et al., 1999). During wakefulness, the hypoventilation can be substantial with PaCO_2_ exceeding that of a control group by more than 10 mmHg (O'Donnell et al., 1999). Replacement therapy by systemic leptin infusion increased minute ventilation as well as the ventilatory response to an inhaled CO_2_ challenge (O'Donnell et al., 1999). The increase in the CO_2_ response of ob/ob mice in response to intracerebroventricular leptin administration strongly argues that at least a component of this response is centrally mediated (Bassi et al., 2012).

The current findings extend this work by directly showing that systemic leptin administration increases respiratory motor output. The underlying mechanism(s) remain to be resolved. O'Donnell et al. (1999) argued that leptin was acting at a CNS site since an increase in metabolism did not appear to be account for the stimulation. However, the metabolic changes in their experiments were complex as the rate of oxygen consumption increased in leptin treated mice although there was no change in carbon dioxide production. This resulted in a decrease in the respiratory exchange ratio from 0.81 ± 0.02 to 0.63 ± 0.02. The current findings extend this earlier work by establishing that the increase in respiratory motor output occurs even when metabolic factors such as PCO_2_ and body temperature are closely controlled (Table 1). In the current study, leptin-induced changes in end-tidal PCO_2_ were prevented by continuously adjusting the CO_2_ level in the inspired gas (see “Materials and Methods”).

The current findings are also consistent with previous work showing that microinjection of leptin into the NTS produces a dose-dependent increase in respiratory motor output of anesthetized rats (Inyushkin et al., 2009; Inyushkina et al., 2010). Inyushkina et al. (2010) attributed the respiratory stimulation, at least in large part, to an inhibition of the reflex termination of inspiration that results from the activation of vagal slowly adapting pulmonary stretch receptors (i.e., the Breuer–Hering inflation reflex) as well as to an increase in the ventilatory response to CO_2_ stimulation of central chemoreceptors (Inyushkina et al., 2010). However, the current study demonstrates that modulation of the Breuer–Hering reflexes is not required for the respiratory stimulation since the vagus nerves were sectioned bilaterally in the neck, thereby eliminating contributions from this and other vagal reflexes. Additionally, the decreased effectiveness of the Breuer–Hering reflexes described by Inyushkina et al. (2010) could arise secondarily to the increased chemoreceptor sensitivity rather than by a direct influence of leptin on the Breuer–Hering reflex. In this regard, increasing PaCO_2_ has been shown to reduce the effectiveness of the Breuer–Hering modulation of respiratory pattern by a mechanism that appears to require input from carotid chemoreceptors (Mitchell et al., 1982; Mitchell and Selby, 1987).

The second mechanism suggested by Inyushkina et al. (2010), an increase in central CO_2_ chemoreceptor sensitivity, is consistent with the tendency for the CO_2_ response curve to become steeper. This is also consistent with the impaired ventilatory responses to CO_2_ seen in leptin-deficient (ob/ob) mice and the recovery of that response following leptin administration either intravenously or into the fourth ventricle (Tankersley et al., 1996; O'Donnell et al., 1999, 2000; Bassi et al., 2012). Whether a change in CO_2_ sensitivity can fully explain the change in ventilation under baseline ventilatory conditions will require further studies to identify the relevant central and/or peripheral chemoreceptors: the key question being whether any leptin-induced change in their discharge can account for the change in phrenic discharge under baseline conditions.

### Effect of systemic leptin infusion on arterial pressure

Leptin administered either systemically or centrally increases sympathetic nerve activity to at least some target tissues (Dunbar et al., 1997; Haynes et al., 1997), and, with chronic administration sufficient to mimic leptin levels seen in obesity, can lead to increases in arterial pressure (Shek et al., 1998). However, acute leptin administration has been reported to increase arterial pressure only when it is administered centrally, an observation explained at least in part by an offsetting increase in nitric oxide production (Frühbeck, 1999; Beltowski et al., 2004). The moderate decrease in arterial pressure in the current study occurred in the absence of a change in heart rate, and is consistent with the action of an additional factor, such as the production of nitric oxide, that decreases the total peripheral resistance. However, the dose of leptin used in the current study is similar to that used by others who did not observe a decrease in arterial pressure (Haynes et al., 1997; Frühbeck, 1999). The reason for the relatively greater impact of a factor such as nitric oxide in the current study is unclear and merits further investigation.

## Summary and interpretation

Systemic administration of leptin led to a progressive increase in the peak amplitude of phrenic nerve bursts. The time course and pattern of the leptin-induced increase in phrenic motor output is very similar to the pattern of plasticity (long-term facilitation; Feldman et al., 2003) in phrenic nerve output that follows either repeated brief periods of electrical stimulation of the carotid sinus nerve or repeated brief exposures to hypoxia (Feldman et al., 2003). Whether the phrenic response to leptin is a form of plasticity depends on whether there is an increase in leptin at the relevant receptors at the end of the 2 h recording period, i.e., 60 min after the end of leptin infusion. Hill et al. (1998) have described the disappearance of leptin with 2 half-lives of 71 and 3.4 min. The longer half-life is the result of endogenous leptin binding to a plasma carrier molecule as well as tissue binding sites. Injections of larger doses of exogenous leptin would be expected to saturate these binding sites and the unbound component would be subject to degradative mechanisms that would result in the shorter (3.4 min) half-life. On this basis, our infused leptin would be expected to largely decline to “physiological” levels after the end of the infusion period. It should also be noted, that since leptin access to the brain is largely via a receptor-mediated mechanism; access to the brain should also be limited throughout the experiment. Nevertheless, if the bound pool is sufficient to elicit the increase in phrenic nerve discharge, longer follow-up times will be needed to determine whether leptin induces plastic increases in the respiratory motor output.

In behaving mammals, an increase in respiratory drive is important for arterial blood gas homeostasis in the face of a leptin-induced increase in energy expenditure (Morton and Schwartz, 2011). In humans, a deficiency in leptin is rare, but a subset of obese patients appears to develop “leptin resistance” (Campo et al., 2007; Piper and Grunstein, 2011). Consistent with animal studies, a subset of obese individuals have substantially elevated circulating levels of leptin and exhibit a decreased respiratory drive and CO_2_ sensitivity. They also have a high incidence of obstructive sleep apnea and obesity hypoventilation syndrome. The latter consists of daytime hypoventilation with hypercapnia and hypoxemia (Piper and Grunstein, 2011). Interestingly, their elevated leptin levels correlate better with hypercapnia than with adiposity (Phipps et al., 2002). Reduced central concentrations of leptin, due to defective leptin transport, or defects in the signal transduction pathways activated by leptin could account for the similarities between the respiratory profile of obese individuals with leptin resistance and that of the *ob/ob* mice.

Taken together with the earlier literature, these data suggest that leptin has an important role in matching ventilation to the metabolic requirements imposed by adipose tissue. The mechanisms by which PaCO_2_ is maintained within narrow limits despite marked changes in metabolism are largely a mystery. Our results demonstrating leptin modulation of breathing begin to address this question. Further elucidation will require identification of the central pathways and molecular mechanisms by which activation of central leptin receptors alters breathing.

### Conflict of interest statement

The authors declare that the research was conducted in the absence of any commercial or financial relationships that could be construed as a potential conflict of interest.
